# Validation of the Dutch Eating Behavior Questionnaire in a Romanian Adult Population

**DOI:** 10.3390/nu13113890

**Published:** 2021-10-29

**Authors:** Lidia Iuliana Arhire, Otilia Niță, Alina Delia Popa, Ana-Maria Gal, Oana Dumitrașcu, Andreea Gherasim, Laura Mihalache, Mariana Graur

**Affiliations:** Faculty of Medicine, “Grigore T. Popa” University of Medicine and Pharmacy, 700115 Iași, Romania; lidia.graur@umfiasi.ro (L.I.A.); alina.popa@umfiasi.ro (A.D.P.); oana.dumitrascu1@gmail.com (O.D.); andreea.gherasim@umfiasi.ro (A.G.); laura.mihalache@umfiasi.ro (L.M.); graur.mariana@gmail.com (M.G.)

**Keywords:** DEBQ, emotional eating, external eating, dietary restraint, assessment of eating behavior, obesity causes, matched treatment obesity

## Abstract

(1) Background: Obesity, part of the triple global burden of disease, is increasingly attracting research on its preventive and curative management. Knowledge of eating behavior can be useful both at the individual level (to individualize treatment for obesity) and the population level (to implement more suitable food policies). The Dutch Eating Behavior Questionnaire (DEBQ) is a widely used international tool to assess eating behavior, i.e., emotional, external and restricted eating styles. The aim of this study was to validate the Romanian version of DEBQ, as obesity is a major concern in Romania. (2) Methods: Our study tested the psychometric properties of the Romanian version of DEBQ on an adult population and explored the associations of eating behavior with weight status (3) Results: The study showed a factor load similar to the original version of the questionnaire and a very good internal validity (Cronbach’s alpha fidelity coefficient greater than 0.8 for all scales of the questionnaire) for the Romanian version of DEBQ and showed that all of the scales positively correlated with body mass index in both men and women. (4) Conclusions: This study will enable the use of the DEBQ Romanian version on the adult population of Romania where the findings could be incorporated into developing better strategies to reduce the burden of nutrition-related diseases.

## 1. Introduction

Obesity has reached pandemic proportions and is currently considered a threat to public health [1]. The prevalence of obesity and associated metabolic diseases is continuing to increase globally [2,3]. Obesity is currently the most significant form of malnutrition, affecting both the developed and the developing world, but significantly more in lower income countries and subpopulations [4]. There are many pathways to obesity—some arising from lifestyle [5] but also from metabolic risk factors [6] with complex interactions between genetic, physiological, psychological and social factors [7]. The description of a single defective control mechanism for food consumption in people with obesity is not an achievable goal [8,9]. Physiological mechanisms such as hunger and satiety [10] are dependent on homeostatic feedback, but also environmental, hedonic feedback, in relation to biological and psychological learning mechanisms (retention/disinhibition) [11,12,13]. Disorders of these regulations, either positive or negative, attract somatic but also psychological complications, which require medical control and adequate scientific nutritional advice. Permanent self-regulation failures can lead to overweight and obesity [14].

The description of obesity treatment postulated by Stunkard more than half a century ago seems to remain valid even today: “The most obese people will not follow a treatment for obesity. Among the people who will follow the treatment, some will not lose weight, and among those who will lose weight over time, they will regain their weight” [15].

Despite numerous researches claiming obesity as a chronic disease with complex (yet incomplete known) mechanisms and numerous prevention and treatment guidelines, too many people continue to follow various fad diets. Many people with obesity cannot maintain medical recommendations [16,17], and the failure of restrictive diets is already known [18]. Usually after losing 10% of body weight, a person will regain most of it within 1 year and all of it in 5 years [19].

There is a discrepancy between the knowledge gained about the importance of a healthy lifestyle and prudent eating patterns to prevent disease, and the awareness of this relationship and the social actions taken to optimize nutritional status [20]. Therefore, efforts to reduce and/or maintain body weight are disappointing and attest to the difficulty/inefficiency of internalizing behavioral principles [21]. Nutritional assessment, which includes behavioral assessment, seems to be the key. There is an important difference between food intake and food behavior, but in order to understand/manage obesity efficiently, it is important to perform accurate and scientific assessment of both [22]. Moreover, in terms of eating behavior, a comprehensive approach to understand environmental influences, food preferences or a certain relationship with food should be the norm. That is why classical techniques of psychological measurement (psychometrics) reached the field of obesity research. It has been postulated that people with obesity differ in terms of eating behavior and the focus of treatment should be on improving it [23]. In this context, questionnaires have proved to be reliable and valid tools for assessment, as long as their construction had been scientifically correct and they are used for the purpose created. Three main psychological theories on overeating have proved their worth, namely: the psychosomatic theory [24], the theory of externality [25] and the theory of restriction [26]. Psychosomatic theory refers to emotional eating (overeating in response to distress (negative emotions, emotional arousal, such as anger or anxiety) [27]. The theory of externality focuses on overeating in response to food-related stimuli (appearance, taste, smell) unrelated to hunger [28]. Restriction theory is based on the effect of disinhibition: people on a diet (“restraint consumption”) when they decrease their cognitive decision to eat less, eat more than normal in response to influences such as distress or pre-loading [29,30,31]. As psychological theories, both externality and psychosomatic theory have a well-established role in the development of obesity, even if causality is difficult to sustain [32]. However, it was the development of psychometric assessment instruments that connected the gap between medical sciences (nutrition, epidemiology) and behavior science, and human food behavior could be better understood [11,13,32]. Using psychometric assessment instruments in clinical practice leads to a more patient-centered (individualized) approach to obesity management: for example, providing more psychological support to those who experience emotional eating [27,33], incorporating mindfulness techniques for those with external eating [34], or using restriction wisely as a strategy for weight loss [35]. Additionally, as behavioral factors influence food choice, but are also themselves influenced by the environment [7], using psychometric assessment instruments in epidemiological studies facilitate the approach of the problem of obesity using an ecological model [7].

Comprehensive assessment of eating behaviors is difficult to perform [11] and the quality of the information collected depends on the quality of the methods used. The content of all the theories quoted above is found in the Dutch Eating Behavior Questionnaire (DEBQ) developed in 1986 [36] validated on the Dutch population for all its components [37,38,39,40]. Shortly after publication, the English version was also validated [32]. Moreover, DEBQ has been validated on different populations: Maltese [41], Italian [42], Spanish [43,44], Chinese [45], French [46,47], Polish [48] German [49], on people with obesity [50] and on twins [51]. DEBQ has been used as a reliable assessment tool, with a robust structure that provides consistent information, adequate for the target population (adults/children, women/men, normal weight/obesity, from various cultures) and which has proved valid for the specific purpose for which it was created. It has a good internal consistency measured in multiple studies and variate settings by consistent statistical operations (Cronbach’s coefficient alpha for reliability, analysis of its internal structure using exploratory structural equation modelling) [39,52,53,54].

Romania is a country with an increased prevalence of overweight people and obesity (31.1% and 21.3%, respectively, of the adult population) [55] and a very high prevalence of type 2 diabetes mellitus, 11.6% [56], these numbers being higher than the European averages [2]. Therefore, it is useful to have a better understanding of eating behavior in Romania so that the problem of obesity is tackled both at the level of prevention of the disease (through policies to improve the food environment) and at the clinical level (for a more individualized approach of obesity treatment). At the moment, there are few studies exploring eating behavior of Romanians.

As DEBQ has proved over the years to be an essential tool for the study and management of obesity in many European countries [40,41,42,43,46,49], it would be a valid quest to use it in the Romanian population. However, when aiming to use an internationally validated questionnaire, it is important to have an accurate and culturally appropriate use of terms. That is why, more important than the technical problem posed by the perfect translation of the original document is the problem of the possibilities of translation and application of the cultural concepts that led to the elaboration of the original questionnaire [57]. Therefore, the main objective of this study was to validate the DEBQ in Romanian.

## 2. Materials and Methods

### 2.1. DEBQ Translated and Used in Romanian

The standard stages for the “cross-cultural” validation of the questionnaire were used in this study: double parallel and independent translation (by a professional translator and one member of the research team), double reverse translation (by other translators than those who performed the first translation), arbitration of differences during a consensus meeting, carrying out pre-tests with questions from the questionnaire and final changes in order to obtain the final version of the questionnaire which was used in this study. The following steps were to apply the questionnaire to the study population, to score the results obtained from the application of the questionnaire and to use the statistical methods for validating the questionnaire.

The Romanian version of DEBQ has the same 33 questions as the original version. The items are grouped into 5 scales, a general scale for emotional eating (Scale A, with 13 questions), but also 2 separate scales for emotional eating, one that responds to diffuse emotions (Scale B, 4 questions) and the other that responds to clearly defined emotions (Scale C, 9 questions). Scale A is the sum of Scale B and C. Scale D is generated by 10 questions and represents eating caused by external factors, whereas Scale E (10 questions) represents “restrained eating”. All of the questions have 5 answer choices, with scores from 1 to 5 (1 being “never” to 5 being “very often”). According to the instructions for use from the original questionnaire, the scores for questions of each scale were added to create a raw score, which was then divided by the number of items per scale to generate the score for each scale. In order to interpret the results, the reference tables with the average scores from the original study were used [58]. In order to translate and use DEBQ in Romanian, permission was obtained from the publisher of the questionnaire, Hogrefe Uitgevers, Amsterdam, Nederland.

### 2.2. Study Population and Collection of Data

This study was conducted on healthy adult volunteers (aged 18 years or over) from our university center during 2020–2021, both onsite and online (due to the pandemic restrictions). As DEBQ has an introductory part, apart from answers to the 33 questions, all of the data required was collected, i.e., demographic data (age, sex), anthropometric data: current weight and height (which were self-reported, expressed in kilograms and meters respectively), data on the evolution of weight, weight gained or lost in the last 6 months and which was the highest and lowest weight. The anthropometric data collected were used to calculate body mass index (BMI). BMI values were used to define overweight and obesity in accordance with standards developed by the International Obesity Task Forces (IOTF) and recommended by the World Health Organization (WHO), as follows: underweight: <18.5 kg/m^2^, normal weight: 18.5–24.9 kg/m^2^, overweight: 25–29.9 kg/m^2^, obesity: ≥30 kg/m^2^ [59]. Only participants who answered all items of the questionnaire were included in the final analysis.

### 2.3. Statistical Analysis

All data collected from the study were recorded in a database created using Microsoft Excel, version 2010. The discrete parameters were numerically recoded and noted in the database with these codes. SPSS v.20 was used for the statistical analysis of data.

Continuous variables were expressed as mean ± standard deviation (SD), and discrete variables as number and proportions. For the analysis of the correlations between the linear parameters, the Pearson method was used for the parameters with normal distribution and the non-parametric Spearman form for those with abnormal distribution (the data distribution was verified with the Kolmogorov test).

In order to perform factor analysis on the structure of DEBQ, firstly the Kaiser-Meyer-Olkin (KMO) test was performed, to test that the data was suitable for factor analysis. The scores for the 33 items of the questionnaire were then analyzed using the Varimax method of principal component analysis to confirm that the grouping of questions on the separate scales from the original questionnaire was maintained in the Romanian translation. Only factors with an Eigenvalue of >1 were retained. Reliability of the questionnaire was assessed using Cronbach’s alpha fidelity coefficient; a coefficient of >0.7 proves the reliability of the questionnaire.

T-student test or ANOVA test (for more than two categories) were used for the comparison of continuous variables; when the data did not show homogeneity of variances, nonparametric rank comparison tests were used: Mann-Whitney test for the comparison of two data sets and the Kruskall Wallis test for the analysis of more than 2 categories of variables. Chi square (χ^2^) was used for comparison of categorical data (for χ^2^ the significance threshold was *p* = 0.1). Testing of the statistical hypotheses was performed with a confidence interval of 95%, resulting in a significance threshold *p* = 5% = 0.05.

Pearson’s correlations (or Spearman’s correlations when the data imposed a nonparametric test) were used to evaluate correlations between the score obtained for each scale of DEBQ and age and BMI of participants.

### 2.4. Ethical Considerations

The study received the approval of the Ethical Committee of our University (approval no. 2/27.07.2020) and participants were included in the study only after they gave informed consent.

## 3. Results

There were 503 participants in the final analysis, among which 76.7% (*n* = 386) were women. The average age of the participants was 30.3 ± 9.7 years, with no significant differences between men and women. The average BMI of men (26.9 ± 4.5 kg/m^2^) was significantly higher (*p* < 0.001) than the BMI of women (23.6 ± 4.4 kg/m^2^). The average BMI in the study population was 24.2 ± 4.6 kg/m^2^. According to the categories of BMI, only 5.6% (*n* = 28, all women) were underweight, 59.0% (*n* = 297, 41 men and 256 women) were of normal weight, 23.3% were overweight (*n* = 117, 49 men and 68 women) and 12.1% (*n* = 61, 27 men and 34 women) presented obesity. For further analysis, the participants were divided into individuals with excess weight (overweight or obesity) and individuals without excess weight (taking together participants with normal weight and those underweight) (data presented in Supplementary Materials Table S1).

The Kaiser-Meyer-Olkin (KMO) test had a value of 0.993, confirming that data was suitable for factor analysis. A total of 5 factors had an Eigenvalue >1 and were retained for the analysis. The Varimax method of principal component analysis showed that factor loadings for the 33 items respected the same distribution as the grouping of questions on the scales of the original version of DEBQ (data presented in Supplementary Materials Table S2).

The Cronbach’s alpha coefficient was calculated for each scale. This was above the appropriate consistency threshold of 0.7 for each scale; more precisely, it was 0.954 for Scale A, 0.84 for Scale B, 0.953 for Scale C (the scales which tested for emotional eating), 0.856 for Scale D (eating in response to external stimuli), and 0.913 for Scale E (eating as a consequence of restraint). Thus, the five scales of the applied DEBQ version proved an adequate internal consistency (data presented in Supplementary Materials Table S3).

Table 1 presents the average scores obtained for each scale of DEBQ by the participants, separately for men and women and in the total study group, the significant differences in the scores of men vs. women being highlighted (obtained in all scales except Scale D). However, as stipulated in the original version of DEBQ, the interpretation of the scores in men and women is different. Therefore, the values from the reference tables were used to evaluate the percent of men and women respectively who obtained a score higher than average in this study. The findings (scores higher than average for gender) were: for Scale A, 45.3% (*n* = 53) of men and 48.7% (*n* = 188) of women; for Scale B, 37.6% (*n* = 44) of men and 32.1% (*n* = 124) of women; for Scale C, 47% (*n* = 55) of men and 48.4% (*n* = 187) of women; for Scale D 62.4% (*n* = 73) of men and 54.4% (*n* = 210) of women and for Scale E, 23.9% (*n* = 28) of men and 42.5% (*n* = 164) of women. There was a significant difference in these numbers only for Scale E (restraint eating), where the percentage was significantly higher in women.

Age positively correlated only with the scores obtained on Scale E (restrained eating), both in men (r^2^ = 0.33, *p* < 0.001) and in women (r^2^ = 0.237, *p* < 0.001). BMI correlated positively and with statistical significance with the scores in each scale in men (for Scale A, r^2^ = 0.271, *p* = 0.003, for Scale B, r^2^ = 0.359, *p* < 0.001, for Scale C, r^2^ = 0.204, *p* = 0.027, for Scale D, r^2^ = 0.353, *p* < 0.001 and for Scale E, r^2^ = 0.215, *p* = 0.02). In women, the significant positive correlations with BMI were found with the scores in all scales except for scale D (external eating): for Scale A, r^2^ = 0.258, *p* < 0.001, for Scale B, r^2^ = 0.207, *p* < 0.001, for Scale C, r^2^ = 0.261, *p* < 0.001, for Scale E, r^2^ = 0.192, *p* < 0.001). By dividing the population by the absence or presence of excess weight, there were significantly higher scores in men with excess weight for Scale B (emotional eating, diffuse emotions), Scale D (external eating) and E (restraint eating). In women, for all scales with the exception of Scale D, the scores of women with excess weight were significantly higher. These data are presented in Table 2.

## 4. Discussion

The Dutch Eating Behavior Questionnaire has the advantage of assessing together three distinct eating behaviors in adults [31] and has very robust psychometric properties that have been proved in a large number of studies over the years [60]. This study showed the same adequate internal consistency of the Romanian version of DEBQ: The Cronbach’s alpha coefficient was excellent for emotional eating scale general and specific (0.954 for Scale A, 0.953 for Scale C) and for restraint eating scale (0.913 for Scale E) and very good for emotional eating scale-diffuse emotions (0.84 for Scale B) and external eating (0.856 for Scale D). The coefficient values of more than 0.8 prove that the questionnaire can be used in clinical practice at individual level [61]. Moreover, in terms of internal structure, the overall results of this study are in line with previous studies [35,40,44,47,62,63]. In this study, the factorial analysis for the Romanian translation of DEBQ showed the presence of the three major factors (‘emotional’, ‘restrictive’ and ‘external’) with same grouping of items as the original Dutch version [58]. The same three-factor structure was found by Wardle in the English validation study [40].

Once food behavior is assessed using DEBQ, personalized therapeutic approaches can be implemented, increasing the chances of success (for the predominance of external factors determining eating behavior, the therapy would aim controlling these stimuli—learn how to eat, avoid places with tempting foods, etc.; for those with the predominance of restraint eating, it would be useful to relearn the physiology of eating—hunger, saturation, satiety and to be in harmony with one’s own weight; emotional eating needs more psychological rather than nutritional support) [58]. It is also important to underline that, even if behavior or diet interventions cannot be appreciated in the same way as drugs in terms of safety and efficacity or in terms of benefits outweighing the risks, they can still have dangerous “side effects”: improper diet or behavioral interventions can exacerbate underlying eating disorders, induce stress and psychological distress, lead to cyclic weight or lower self-esteem [64,65,66]. Therefore, tools used to assess eating behavior should have a solid scientific foundation, and before using an international questionnaire such as DEBQ, it has to be proven that it is culturally appropriate for the intended population and that the psychometrics properties for the specific population are strong.

From a public health point of view, this study shows significant results in terms of possible factors associated with weight gain at population level. The results show that a large proportion of women (42.5% women) scored higher than average on the restraint eating scale. Additionally, the score for restraint eating positively correlated with age and BMI (in both men and women), suggesting that dieting would be a significant risk factor for obesity. This draws attention on the necessity to advocate for medically supervised treatments for obesity and to educate the population to follow healthy eating patterns [67]. Furthermore, a very large proportion of men and women scored higher than average on the external eating scale (62.4% of men and 54.4% of women) and the score for scale D correlated positively with BMI in men. This could be interpreted as a reason to advocate for healthier food environments [54]. Therefore, this study shows that the Romanian version of DEBQ, with its strong psychometric properties, can be used both in clinical setting, in the management of obesity, but also at population level, where the findings could be incorporated into developing better strategies to reduce the burden of nutrition-related diseases.

Some limitations of this research should be mentioned. Our results, based on self-reported information, are susceptible to some respondents’ prejudices and, in addition, we were unable to gather more detailed information about food choice and food intake, in order to compare intention to eat with actual food intake [68]. This would be a perspective for further research. Moreover, the study sample is not representative for Romania, as women are over-represented and data regarding social class or living environment (rural/urban) were not collected, but the results show associations between scale scores and age and BMI which are similar to those obtained in other studies [40,41,44,46,49] which would suggest that the questionnaire can further be used for its purpose in larger Romanian samples.

## 5. Conclusions

Assessing adult eating behavior in a reliable manner (as is the case with validated questionnaires) is an important step in addressing the issue of obesity. Romania is a country with high prevalence of overweight people and obesity and is also lacking solid data regarding food behavior of the population. This study showed that the Romanian version of DEBQ is a reliable and validated tool that can be used both by clinicians and public health specialists.

## Figures and Tables

**Table 1:** Average scores for each scale of DEBQ in the study population.

	Men (*n* = 117)	Women (*n* = 386)	Total (*n* = 503)	*p* Value *
Score for Scale A (mean ± SD) (emotional eating)	1.86 ± 0.74	2.31 ± 0.96	2.21 ± 0.93	<0.001
Score for Scale B (mean ± SD) (emotional eating-diffuse)	2.16 ± 0.86	2.48 ± 0.96	2.4 ± 0.95	0.001
Score for Scale C (mean ± SD) (emotional eating-specific)	1.73 ± 0.75	2.24 ± 1.04	2.12 ± 0.99	<0.001
Score for Scale D (mean ± SD) (external eating)	3.11 ± 0.71	3.05 ± 0.73	3.07 ± 0.72	0.497
Score for Scale E (mean ± SD) (restraint eating)	2.14 ± 0.81	2.76 ± 0.91	2.62 ± 0.93	<0.001

SD = standard deviation; * difference between Men and Women.

**Table 2:** Average scores for each scale of DEBQ in participants with and without excess weight.

	Men			Women		
	without Excess Weight (*n* = 41)	with Excess Weight (*n* = 76)	*p* Value *	without Excess Weight (*n* = 284)	with Excess Weight (*n* = 102)	*p* Value ^#^
Score for Scale A (mean ± SD)	1.7 ± 0.72	1.95 ± 0.75	0.077	2.2 ± 0.9	2.6 ± 1.079	0.002
Score for Scale B (mean ± SD)	1.92 ± 0.7	2.28 ± 0.92	0.030	2.39 ± 0.89	2.71 ± 1.1	0.009
Score for Scale C (mean ± SD)	1.6 ± 0.78	1.81 ± 0.72	0.156	2.12 ± 0.98	2.55 ± 1.13	0.001
Score for Scale D (mean ± SD)	2.83 ± 0.55	3.25 ± 0.74	0.001	3.01 ± 0.73	3.17 ± 0.72	0.073
Score for Scale E (mean ± SD)	1.89 ± 0.74	2.27 ± 0.81	0.012	2.7 ± 0.96	2.94 ± 0.73	0.02

* Difference between Men with and without Excess Weight; ^#^ Difference between Women with and without Excess Weight.

## Data Availability

The data presented in this study are available on request from the corresponding author. The data are not publicly available due to the fact that DEBQ is protected by copyright.

## Supplementary Materials

The following are available online at https://www.mdpi.com/article/10.3390/nu13113890/s1, Table S1: Basic characteristics of the study population, Table S2: Factor loading (Rotated Component Matrix analyzed using Prin-cipal Component Analysis as Extraction Method and Varimax as Rotation Method), Table S3: Reliability Statistics analysis.
