# Supplementary material for: Validation of the Dutch Eating Behavior Questionnaire in a Romanian Adult Population
Source: Nutrients. 2021 Oct 29;13(11):3890. doi: 10.3390/nu13113890 (PMC8619088; doi:10.3390/nu13113890)
Supplement: Supplementary file 1 [file nutrients-13-03890-s001.zip › nutrients-1369274-supplementary.pdf]

Table 1 Supplementary. Basic characteristics of the study population

	Men (n=117)	Women (n=386)	Total (n=503)	p value*
Age (years) (mean±SD)	29.24±10.25	30.69±9.51	30.3±9.7	0.158
Weight (kg) (mean±SD)	84.81±14.48	64.67±12.93	69.35±15.79	<0.001
BMI (kg/m^2) (mean±SD)	26.9±4.5	23.6±4.4	24.2±4.6	<0.001
Subjects with excess weight (number; percent)	76; 65.0%	102; 26.4%	178; 35.4%	<0.001

SD=standard deviation; BMI=body mass index; * difference between Men and Women

Table 2 Supplementary. Factor loading (Rotated Component Matrix analyzed using Principal Component Analysis as Extraction Method and Varimax as Rotation Method)

	Factor 1 (Corresponds to Scale A)	Factor 2 (Corresponds to Scale E)	Factor 3 (Corresponds to Scale D)
Q25	**0.896**	0.079	0.107
Q20	**0.891**	0.077	0.117
Q32	**0.888**	0.126	0.071
Q23	**0.87**	0.153	0.09
Q13	**0.842**	0.079	0.089
Q10	**0.836**	0.065	0.191
Q5	**0.789**	0.152	0.265
Q16	**0.785**	0.102	0.047
Q1	**0.753**	0.102	0.141
Q30	**0.744**	0.096	-0.012
Q8	**0.673**	0.056	0.08
Q28	**0.644**	0.017	0.164
Q3	**0.37**	0.008	0.066
Q22	0.075	**0.858**	0.012
Q26	0.075	**0.843**	-0.021
Q31	0.111	**0.837**	-0.001
Q29	0.095	**0.798**	-0.005
Q7	0.141	**0.771**	-0.078
Q11	0.168	**0.731**	-0.06
Q19	0.091	**0.716**	0.157
Q4	0.01	**0.655**	0.045
Q14	-0.03	**0.612**	-0.069
Q17	0.212	**0.579**	-0.038
Q2	0.212	-0.002	**0.814**
Q15	0.104	0.009	**0.812**
Q33	0.176	-0.041	**0.804**
Q6	0.216	0.008	**0.802**
Q24	0.203	0	**0.717**
Q9	0.073	-0.08	**0.567**
Q21	0.114	0.107	**0.528**
Q12	0.134	-0.094	**0.522**
Q27	0.226	-0.009	**0.398**
Q18	0.185	-0.082	**0.37**

Table 3 Supplementary. Reliability Statistics analysis

	Cronbach's Alpha	Cronbach's Alpha Based on Standardized Items	N of Items
Scale A	0.954	0.954	13
Scale B	0.840	0.840	4
Scale C	0.953	0.954	9
Scale D	0.856	0.857	10
Scale E	0.913	0.912	10
